# Public Perceptions of Climate Change as a Human Health Risk: Surveys of the United States, Canada and Malta

**DOI:** 10.3390/ijerph7062559

**Published:** 2010-06-14

**Authors:** Karen Akerlof, Roberto DeBono, Peter Berry, Anthony Leiserowitz, Connie Roser-Renouf, Kaila-Lea Clarke, Anastasia Rogaeva, Matthew C. Nisbet, Melinda R. Weathers, Edward W. Maibach

**Affiliations:** 1 Center for Climate Change Communication, George Mason University, Fairfax, VA 22030, USA; E-Mails: croserre@gmu.edu (C.R.R); mweathe1@gmu.edu (M.R.W.); emaibach@gmu.edu (E.W.M.); 2 Department of Public Health, Ministry for Health, the Elderly and Community Care, Valletta, VLT 2000, Malta; E-Mail: roberto.debono@gov.mt; 3 Health Canada, Ottawa, K1A 0K9, Canada; E-Mails: Peter.Berry@hc-sc.gc.ca (P.B.); kaila-lea.clarke@hc-sc.gc.ca (K.L.C.); anastasia.rogaeva@hc-sc.gc.ca (A.R.); 4 Yale Project on Climate Change Communication, Yale University, New Haven, CT 06511, USA; E-Mail: anthony.leiserowitz@yale.edu; 5 School of Communication, American University, Washington, DC, USA; E-Mail: nisbet@American.edu

**Keywords:** climate change, global warming, public health, opinion poll, survey, United States, Canada, Malta

## Abstract

We used data from nationally representative surveys conducted in the United States, Canada and Malta between 2008 and 2009 to answer three questions: *Does the public believe that climate change poses human health risks, and if so, are they seen as current or future risks? Whose health does the public think will be harmed? In what specific ways does the public believe climate change will harm human health?* When asked directly about the potential impacts of climate change on health and well-being, a majority of people in all three nations said that it poses significant risks; moreover, about one third of Americans, one half of Canadians, and two-thirds of Maltese said that people are already being harmed. About a third or more of people in the United States and Canada saw themselves (United States, 32%; Canada, 67%), their family (United States, 35%; Canada, 46%), and people in their community (United States, 39%; Canada, 76%) as being vulnerable to at least moderate harm from climate change. About one third of Maltese (31%) said they were most concerned about the risk to themselves and their families. Many Canadians said that the elderly (45%) and children (33%) are at heightened risk of harm, while Americans were more likely to see people in developing countries as being at risk than people in their own nation. When prompted, large numbers of Canadians and Maltese said that climate change can cause respiratory problems (78–91%), heat-related problems (75–84%), cancer (61–90%), and infectious diseases (49–62%). Canadians also named sunburn (79%) and injuries from extreme weather events (73%), and Maltese cited allergies (84%). However, climate change appears to lack salience as a health issue in all three countries: relatively few people answered open-ended questions in a manner that indicated clear top-of-mind associations between climate change and human health risks. We recommend mounting public health communication initiatives that increase the salience of the human health consequences associated with climate change.

## 1. Introduction

Little evidence of the human health impacts of climate change existed in 2001 [1] when the Intergovernmental Panel on Climate Change’s third assessment report was released, but by 2007, the international panel of research scientists announced with very high confidence that “climate change currently contributes to the global burden of disease and premature deaths” [2]. Epidemiological research that until recently had only linked climate change to human injuries, deaths and illnesses resulting from heat waves and infectious diseases is beginning to be augmented by studies that address other potential stressors that may also impact population health, such as refugee migrations and increased vulnerability to poverty [3,4] among others.

Public health officials, at least in some nations, are aware of the growing human health risks associated with climate change. A 2008 survey of Ministries of Health in the British Commonwealth—including Malta’s—found that of the 31 health ministries that responded from 53 member states, all were concerned about climate change’s current or future public health impacts, particularly on children, the elderly and those in poverty, from flooding and sea level rise, changes in temperature and precipitation, and food insecurity [5]. In the United States, a 2008 survey of local public health department directors found that almost 70% believed that their jurisdiction (a county or city) would experience serious negative health effects associated with climate change over the next two decades [6]. Public health officials at all levels—from local to international—are calling for more attention to the issue [7–9].

There has been relatively little research on public awareness and understanding of the human health impacts and risks associated with climate change, and almost none of the research has been published or synthesized in the academic literature. A 2001 survey of the public in 30 countries found that when respondents were provided with a list of potential climate change impacts almost a third of respondents named human health as their greatest concern, the most of any category and higher than droughts and water shortages, extreme weather, and sea level rise [10]. People in developing nations were more likely to cite health impacts as a concern than people in developed nations. Recent polls in Canada also found high rates of identification of climate change as a human health threat when closed-ended questions were used. A survey in 2006 found that 65% of Canadians thought that greenhouse gases would negatively affect health [11], and another in 2007 found 81% were concerned about risks to health associated with climate change [12]. Although not focused primarily on this research question, surveys in the United States have indicated that Americans are less likely to identify climate change as a potential risk to their own health and that of others [13–15]. Therefore, it appears there may be large differences in the ways in which people across different countries respond to survey questions about the risks of climate change to human health, even when prompted.

There have been few cross-national studies on climate change public opinion [16], but even less research is available that compares international perceptions of climate change health impacts. Three national surveys that solely or substantially focused on public perceptions about the health impacts of climate change were conducted recently over a one-year period—February 2008 to 2009—in the United States [17], Canada [18,19] and Malta [20]. While these studies were conducted independently, there was some overlap in the research questions and survey measures due to correspondence between the investigators. The lead investigators in the Canadian (PB) and the American survey (AL and EM) exchanged ideas prior to those surveys being fielded, and one of the American investigators (AL) served as an academic advisor to the Maltese investigator (RD). In this paper, we draw from the data collected in these surveys to answer three broad research questions among members of three developed Western nations:

- RQ1: Does the public believe that climate change poses human health risks? And if so, are they seen as current or future risks?
- RQ2: Whose health does the public think will be harmed?
- RQ3: In what specific ways does the public believe climate change will harm human health?

## 2. Methodology

The three surveys were conducted in 2008 and early 2009 with nationally representative samples of adults in the United States, Canada and Malta. The population samples reflect similar gender divisions and age distributions, but somewhat different proportions of educational attainment (46% of the Canadian sample had a university degree or higher compared to 27% in the U.S. and 17% in Malta) (Table S1, Appendix). All the studies asked questions about respondents’ beliefs, attitudes and behaviors regarding global warming/climate change. The Canadian and Maltese studies specifically addressed the extent of public knowledge and concern about the human health consequences of climate change; the American study addressed this as one of many topics covered in the survey. The American survey employed the term “global warming,” while the Canadian and Maltese studies used “climate change.” Additional crosstabular analysis of the response measures by age, income and education is provided in tables in the Appendix. Further details of study methodology follow below.

### 2.1. United States

This survey was conducted by the Yale Project on Climate Change Communication and George Mason University’s Center for Climate Change Communication using Knowledge Networks’ nationally representative online panel of adults in the United States. Panel members are initially recruited using random digit dialing from a sampling frame of all U.S. phone numbers. Participants are provided hardware and Internet access to enable them to access Web-based questionnaires. The recruitment success rate is approximately 56%. A random sample of these panel members was drawn for the U.S. survey.

To accommodate a large number of survey measures, the U.S. instrument was divided into two questionnaires that were administered between October 7 and November 12, 2008. Of the original 3,997 invited respondents, 2,164 completed both questionnaires, a 54% response rate. The online panel tracks the U.S. Census Bureau’s Current Population Survey (CPS) on demographic variables such as age, race, Hispanic ethnicity, geographic region and employment. In order to adjust for non-coverage or non-response biases, the data was weighted to reflect CPS distributions of age, race, gender and education. The margin for error for the weighted data is ±2% within a 95% probability. Yale and George Mason University’s Human Subjects Review Boards approved the study protocol.

### 2.2. Canada

This survey was conducted by Environics Research Group between February 12 and March 3, 2008, using telephone interviews of 1,600 respondents. The sample of households was chosen using random-digit dialing. Household members 18 years of age or older with the most recent birthday were interviewed, either in English or French. The response rate was 10%. The data was weighted based on the Canadian 2006 Census to reflect regional population demographic characteristics for the country’s 10 provinces and three territories, and by age and gender in line with national population percentages. The final weighted sample under-represented those with lower education levels (25% high school education or less compared to 45% in the 2006 Census). The margin of error for the entire sample is ±2.4% with a 95% probability. Health Canada, which commissioned the research, does not require human subjects approval for public opinion research.

### 2.3. Malta

Using a list-based telephone survey method, this survey was conducted by one of the authors (RD) between January 12 and February 28, 2009. The March 2008 Electoral Register provided the sample frame for the study; phone numbers were identified through the online directories of the two main telephone providers. Interviews were conducted primarily in Maltese (97.4%), with the remaining in English. The sample—stratified by gender, age group, and regional district representative of distributions found within the Maltese adult population—yielded 543 completed questionnaires, a 92.7% completion rate. The final respondent sample was compared to the characteristics of the initial sample of 800 and found to be statistically indistinguishable by age, gender and region. In comparing the sample to the 2005 Census across the categories of labor status, occupation and education, the respondents were slightly more likely to be employed, professionals, and more highly educated. The margin of error is ±5% with a 95% probability. The University Research Ethics Committee provided prior approval of the study protocol.

## 3. Results

### 3.1. Does the Public Believe that Climate Change Poses Human Health Risks? And if so, Are They Seen as Current or Future Risks?

Fifty percent or more of Canadians and Maltese said that climate change is already harming people’s health, while only slightly more than a third of Americans said the same. A majority in all three countries said that in the future climate change will likely cause poverty/reduced standards of living, water shortages, and disease (United States and Malta), and more severe/frequent hurricanes and heat waves (United States and Canada), all of which either directly or indirectly undermine public health.

#### 3.1.1. United States

**Figure 1.**
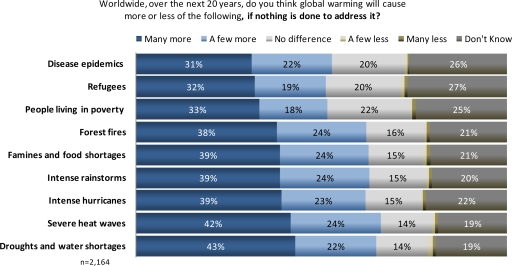
U.S. public perceptions of impacts from global warming.

A majority of Americans said that global warming will cause a range of environmental and societal impacts over the next 20 years (Figure 1). They were more likely to believe global warming will cause more frequent droughts and water shortages (65%), severe heat waves (66%), famines and food shortages (63%), and intense hurricanes (62%) than increases in epidemics (53%), people living in poverty (51%) and refugee migration (51%). About a quarter of respondents said they did not know what the effects of global warming would be (19 to 27%), and slightly fewer said that these events will not increase due to global warming (14 to 22%).

Box 1. **U.S. survey questions addressing if and when global warming will cause health risks.**

- **Worldwide over the next 20 years, do you think global warming will cause more or less of the following, if nothing is done to address it?** Droughts and water shortages, extinctions of plant and animal species, people living in poverty, refugees, disease epidemics, intense hurricanes, floods, forest fires, expanding deserts, melting ice caps and glaciers, intense rainstorms, severe heat waves, famines and food shortages, abandoning large coastal cities due to rising sea levels *[Many more, a few more, no difference, a few less, many less, don’t know]*
- **Now please think about the human health effects of global warming. (Please choose the answer corresponding to your best estimate.) Worldwide, how many people do you think …**
  ○ Currently die each year due to global warming?
  ○ Are currently injured or become ill each year due to global warming?
  ○ Will die each year 50 years from now due to global warming?
  ○ Will be injured or become ill each year 50 years from now due to global warming? *[Millions, thousands, hundreds, none, don’t know]*
- **When do you think global warming will start to harm people in the United States?** *[They are being harmed now, in 10 years, in 25 years, in 50 years, in 100 years, never].*

**Figure 2.**
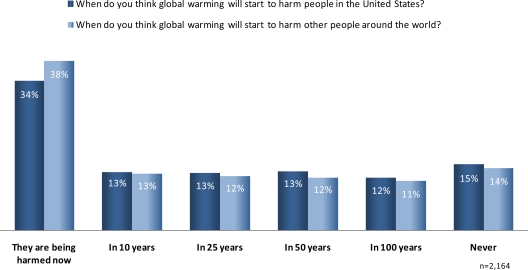
U.S. perceptions of timing of harm to people from global warming.

Sixty percent of Americans thought that global warming will begin to harm people in the United States within the next quarter century (Figure 2). About a third said that people in the United States are already being harmed, and 38% said this is true of people around the world. Only a small proportion—14 to 15%—believed that people will never be harmed by global warming.

**Figure 3.**
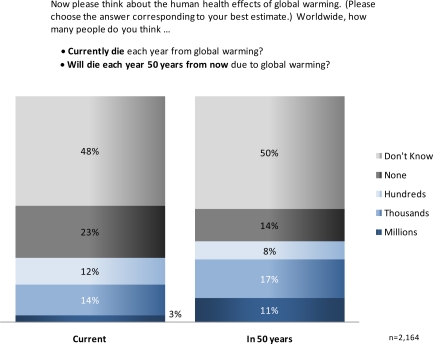
U.S. perceptions of current and future deaths resulting from global warming.

**Figure 4.**
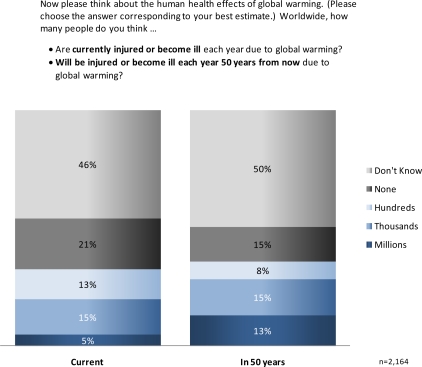
U.S. perceptions of current and future illnesses and injuries resulting from global warming.

Americans had difficulty estimating the number of people worldwide who are currently—or will be in the future—harmed by global warming. Almost half responded they do not know whether hundreds, thousands or millions of people are currently being injured/becoming ill (46%) or dying (48%) as a result of global warming (Figures 3 and 4), and slightly more indicated “don’t know” regarding these health impacts in the future (50% injuries/illness; 50%, deaths). Of those who were willing to estimate current health impacts, a plurality estimated “none” (21%, injuries/illness; 23%, deaths), followed by thousands (15%, injuries/illness; 14%, deaths), hundreds (13%, injuries/illness; 12%, deaths) and millions (5%, injuries/illness; 3%, deaths). Larger numbers of people estimated there would be injuries and deaths in 50 years as a result of global warming: millions of future injuries (13%), and thousands (17%) or millions (11%) of future deaths. Only 14 to 15% of Americans foresaw no harm to people worldwide by the next half century.

#### 3.1.2. Canada

Box 2. **Canada survey questions addressing if and when climate change will cause health risks.**

- **Would you say that climate change definitely causes, likely causes, likely does not cause or definitely does not cause each of the following types of environmental impacts in Canada?** Heat waves, more frequent storms including hurricanes, drought conditions, flooding of rivers/coastal areas, extreme cold weather, melting permafrost in Arctic, loss of wildlife habitat, coastal erosion, forest fires *[Read only 6 of 9 items to reduce burden of response]*
- **For each of these potential risks to health, would you say the risks to Canadians have generally increased, have generally decreased, or remained the same over the past ten years or so?** Heat waves, more frequent storms including hurricanes, drought conditions, flooding of rivers/coastal areas, extreme cold weather, melting permafrost in Arctic, loss of wildlife habitat, coastal erosion, forest fires *[Read only 6 of 9 items to reduce burden of response]*
- (Unprompted) **In your view, what environmental problem or hazard would you say poses the greatest risk to the health of Canadians?**
- **I will now read you a list of potential risk to the health of Canadians. Please tell me whether you think each of the following poses a major risk, a moderate risk, a minor risk, or no risk at all to the health of Canadians.** Second-hand smoke from tobacco, chemical pollution, climate change, air pollution, heat waves, obesity, heart disease, pesticides in food, pandemic flu epidemics, West Nile virus, extreme cold weather, tap water *[Read only 8 of 12 items to reduce burden of response]*
- **Do you think that climate change already poses a risk to Canadians today, or do you think this is something that will happen in the future?**
- (Asked if respondents answer either “depends” or “in the future” to the above question) **Do you think climate change will start affecting the health of Canadians:** [in the] next 5 years, next 6–10 years, next 11–25 years, at least 25 years from now, never, depends, don’t know, not applicable.

**Figure 5.**
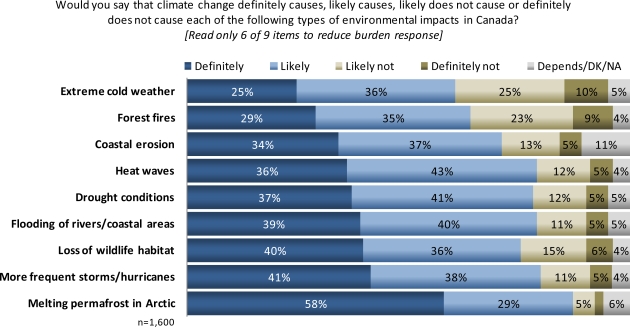
Canadian perceptions of environmental impacts resulting from climate change.

When asked in an open-ended question what environmental problem or hazard poses the greatest risk to their nation’s public health, only 10% of Canadians named climate change, while 54% cited air pollution/smog and 18% cited water pollution. When specifically prompted, a large majority said that climate change is likely to trigger environmental conditions that are harmful to human health (61–87%, Figure 5). Similar to Americans, sizable numbers of Canadians said that climate change likely or definitely causes more frequent storms/hurricanes (79%), flooding of rivers and coastal areas (79%), heat waves (79%), and drought conditions (78%).

#### 3.1.3. Malta

Box 3. **Malta survey questions addressing if and when climate change will cause health risks.**

- **How likely do you think it is that each of the following will occur during the next 50 years due to climate change?**
  ○ Worldwide, many people’s standard of living will decrease due to climate change.
  ○ Worldwide, water shortages will occur due to climate change.
  ○ Increased rates of serious disease worldwide due to climate change.
  ○ You or your family’s standard of living will decrease due to climate change.
  ○ Water shortages will occur in Malta due to climate change.
  ○ The chance of you or your family getting a serious disease will increase due to climate change.
    [Very unlikely, somewhat unlikely, somewhat likely, very likely, don’t know]
- **Do you think people can die because of climate change?** *[Yes, no, don’t know]*
- **If yes, worldwide, do you think this is happening now or is it something that will happen in the future?** *[Now, future, don’t know]*
- **Do you think people can become ill because of climate change?** *[Yes, no, don’t know]*
- **If yes, worldwide, do you think this is happening now or is it something which will happen in the future?** *[Now, future, don’t know].*

When asked to classify a series of threats posed to the health of Canadians as being major, moderate, minor or no risk, 32% ranked climate change as a major risk, below obesity (70%), heart disease (65%), and air pollution (62%), but above pandemic flu epidemics (29%), heat waves (20%) and West Nile virus (16%).

More than half of Canadians (54%) identified climate change as a current risk to the health of their nation’s citizens as opposed to a future threat (39%). Very few said climate change will never pose health risks (4%) with about the same number (3%) saying that it depends/don’t know/not applicable. Of those who reported health impacts will not be felt until the future or that it depends, 40% said health impacts will begin within the next 10 years, 29% said health impacts will begin in 11–25 years, and 22% said 25 years or more.

Large numbers of Canadians said that health risks to Canadians from climate change (69%) and related problems including air pollution (76%), West Nile virus (50%), and heat waves (48%) have been increasing over the past decade.

**Figure 6.**
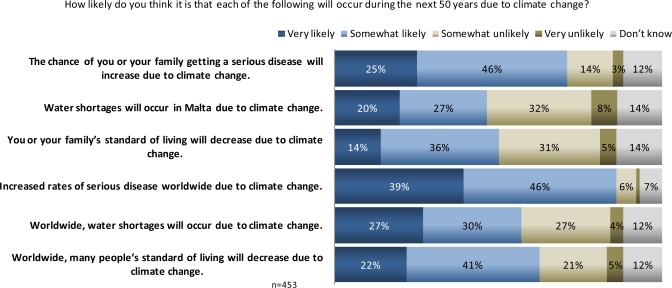
Maltese perceptions of likelihood of health risks resulting from climate change.

About half or more (47–85%) of Maltese said that climate change over the next half century will cause declines in people’s standard of living, and increases in rates of serious disease and water shortages (Figure 6). Increasing rates of serious disease were seen as the most likely consequence of climate change of the options presented (worldwide, 85%; you/your family, 71%), followed by declines in standard of living (worldwide, 63%; you/your family, 50%). Water shortages were least likely to be identified as a climate change impact both worldwide (57%) and in Malta (47%).

Compared to Americans, a much larger proportion of the Maltese said that climate change is already causing death and illness worldwide. Nearly all (89%) Maltese said that climate change can cause illness, with nearly two-thirds (63%) saying that it is happening now. Somewhat fewer said that people can die because of climate change (77%), with half (50%) saying that this is already happening now. Only small percentages said they do not believe climate change can cause people to die (11%) or become ill (6%), and similarly small percentages said they “don’t know” (12% and 5%, respectively).

### 3.2. Whose Health does the Public Think will be Harmed?

Less than half of Americans said that they believe they themselves, or those people close to them, will be harmed by global warming; they were more likely to cite people in developing countries and future generations as those who are most at risk. Conversely, between about one-half to three-quarters of Canadians said that they themselves, their community, and their family are vulnerable to the human health impacts of climate change, and about equal numbers of Maltese were concerned about climate change’s impacts on people all over the world, and on themselves and their families.

#### 3.2.1. United States

Box 4. **U.S. survey questions addressing whose health will be harmed by global warming.**

- **How concerned are you about the impact of global warming on … ?** All people, all children, your children, people in the United States, you, your health, your lifestyle, your future *[Scale from 1=not at all concerned to 7=extremely concerned]*
- **How much do you think global warming will harm … ?** You personally, your family, your community, people in the United States, people in other modern industrialized countries, people in developing countries, future generations of people *[A great deal, a moderate amount, only a little, not at all, don’t know].*

**Figure 7.**
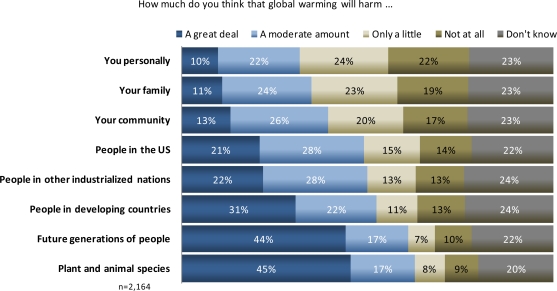
U.S. perceptions of who will be the most harmed from global warming.

Americans were far more likely to see climate change as a problem for people geographically and temporarily distant, rather than for themselves (Figure 7). A majority of respondents said that global warming will harm future generations (61%) and people in developing countries (53%) a great deal or moderate amount. Conversely, far fewer respondents said global warming will harm themselves (32%), their family (35%), or their community (39%) to the same degree.

Americans on average said that they were more concerned about the effects of global warming on human beings than not. On a scale from not at all concerned (1) to extremely concerned (7), they indicated the most concern for all children (*M* = 5.01), all people (4.79) and their children (4.78) and the least amount of worry about global warming’s impacts on their own lifestyle (4.03).

#### 3.2.2. Canada

Box 5. **Canada survey questions addressing whose health will be harmed by climate change.**

- **What about your own health? Do you believe that you personally are definitely, likely, likely not, or definitely not vulnerable to the potential health impacts of climate change?**
- **Do you believe that people living in your community are definitely vulnerable, likely vulnerable, likely not vulnerable, or definitely not vulnerable to the potential health impacts of climate change?**
- (Open-ended) **What types of Canadians, if any, do you think might be most likely to experience the negative effects of climate change?**

Canadians said that the elderly (45%), children (33%) and people with illnesses (14%) will be the most likely to experience the negative effects of climate change. Though Canada is an Arctic nation with long coastlines, relatively few said people in the North/Arctic (8%) and those living near oceans/coasts (5%) would be especially vulnerable. Two thirds reported feeling personally vulnerable to the potential health impacts of climate change (67%) and almost one half (46%) said someone in their immediate household is especially vulnerable. Additionally, a large majority of Canadians viewed their community as being definitely or likely vulnerable (76%).

#### 3.2.3. Malta

Box 6. **Malta survey questions addressing whose health will be harmed by climate change.**

- **Which of the following are you most concerned about? The impacts of climate change on …?** You and your family, the Maltese people, people all over the world, non-human nature, not at all concerned.

About a third of Maltese said they were most worried about the impacts of climate change on themselves and their families (31%), another third identified other people around the world (32%), and a quarter indicated that they were most worried about non-human nature (26%). Only a small proportion of respondents said they were most concerned about impacts on the Maltese people (5%) or that they were not at all concerned (6%).

### 3.3. In What Specific Ways does the Public Believe Climate Change will Harm Human Health?

When presented with a list of potential health risks from climate change, the majority of people in both Canada and Malta said the changing climate will cause increased respiratory and breathing difficulties, cancer and heat-related health problems. No questions addressed this topic in the United States survey.

#### 3.3.1. Canada

Box 7. **Canada survey questions addressing types of health conditions affected.**

**Canada:**

- (Open-ended) **I would now like to ask you about how climate change may affect the health of Canadians. In what ways, if any, do you think climate change poses a risk to the health of Canadians?**
- **I will now read you a list of health risks that affect many Canadians today. Would you say that climate change definitely, likely, likely not or definitely does not increase the risk of:** cancer, heat stroke, respiratory/breathing problems, infectious diseases, injuries from storms/extreme weather events, sunburn.

**Figure 8.**
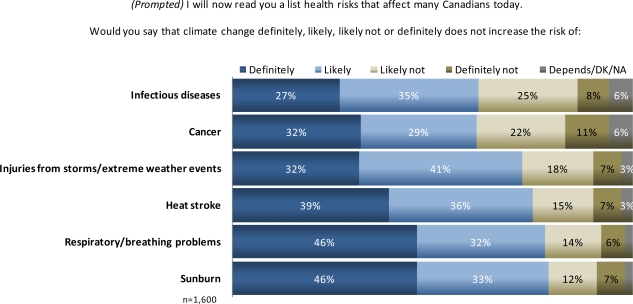
Canadian perceptions of likelihood of increased specific risks from climate change.

In the Canadian survey, respondents were first asked without prompting to identify one or more health conditions that are impacted by climate change. Sixty percent could name at least one, but the answers were exceedingly diverse, with low percentages of responses in any one category (<22%). In response to the open-ended question “In what ways, if any, do you think climate change poses a risk to the health of Canadians?” the most common answers were respiratory/breathing problems (22%), infectious diseases (11%), cancer (11%), and air quality impacts (8%). One of the most commonly cited health risks from climate change, respiratory or breathing problems, was mentioned unprompted by less than a quarter of Canadians, but when asked specifically almost 80% said they thought it was a definite or likely consequence (Figure 8). Indeed, when prompted with a list of potential conditions that may be affected, a large majority of Canadians (62–79%, Figure 8) cited multiple health consequences from climate as definitely or likely. This difference in responses between prompted and unprompted questions may be an indication that climate change health risk information is still relatively new for the public and either unknown or less cognitively salient.

#### 3.3.2. Malta

Box 8. **Malta survey questions addressing types of health conditions affected.**

**Malta:**

- **The following list contains items, some of which are affected by climate change while others are not. Which of the following is affected by climate change?** Heat waves, skin cancer, infections which can cause diarrhea, cardiovascular conditions, allergies, infectious diseases such as malaria, asthma and respiratory conditions.

**Figure 9.**
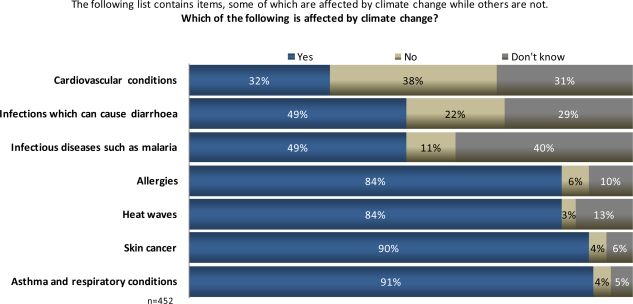
Maltese public perceptions of types of health risks from climate change.

Large percentages of Maltese identified asthma and respiratory difficulties (91%), skin cancer (90%), heat wave events (84%), and allergies (84%) as being associated with climate change (Figure 9). Only about a third identified cardiovascular problems as a result of climate change, and just less than half said a changing climate will cause more infectious and diarrheal diseases.

## 4. Discussion

In this paper, we have attempted to synthesize findings from three largely independent representative national surveys conducted in three distinctly different, albeit developed Western nations. That said, we urge appropriate caution in interpreting our findings due to limitations in the methods. While each of the surveys used was methodologically sound, synthesizing their findings to answer a set of overarching research questions is inherently limited by differences in measures, research questions, and foci (e.g., while the Canada survey focused solely on perceived health impacts for Canadians, the U.S. and Malta surveys also assessed perceived impacts on people elsewhere in the world). Research has indicated that even the use of the term global warming as opposed to climate change, as was done in these surveys (global warming in the U.S. survey, and climate change in Malta and Canada’s), may impact survey responses [21,22]. We have presented a range of detailed results, but will limit our discussion to the big picture findings that emerge from the data.

### RQ1: Does the public believe that climate change poses human health risks? And if so, are they seen as current or future risks?

Substantial numbers of the American, Canadian and Maltese people appear to believe that climate change poses important risks to human health and well-being now or in decades to come. With regard to timing, about one third of Americans believe that health impacts are already occurring, while about half of Canadians and two thirds of Maltese believe that people are being harmed now.

The most commonly perceived threats to health and well-being, however, differed between countries. For example, Americans saw droughts and water shortages as one of the most “likely” global warming impacts (65%), the Maltese saw it as the least likely (where water shortages in Malta and worldwide were seen as “likely” by 47% and 57%, respectively), and Canadians placed it in the middle of the range of likely impacts, although they were more apt to see it as a likely risk than Americans (drought conditions, 78%). These differences are probably due in part to the array of response options provided in each of the surveys. Cultural differences, local and regional climate conditions, and personal experiences may also have played a role in these differences. Many regions of the United States and Canada have experienced drought conditions over the past decade [23], which may influence perceptions. Malta is unique in this respect as it has the capacity to supply demand for potable water by desalinating sea water.

Even by the early 1990s, concern over climate change was highest internationally in Canada, Europe and South America, and lower in the United States [15]. American concern over global warming has continued to rank lower compared to other nations, in part due to the larger representation of climate change deniers [24]. Standardized questions about health impact should be developed so that future surveys can more meaningfully explore these perceptions within and between regions and nations, and particularly address potential differences between developed and developing countries.

It is important to note, however, that climate change may lack salience as a health issue in the three countries studied. When asked closed-ended questions, many respondents gave answers consistent with beliefs in climate change as a threat to human health. Conversely, when asked open-ended questions, and closed-ended questions of a more specific nature, relatively few respondents gave answers consistent with perceptions of climate change as a serious risk to human health. For example, few Canadians, unprompted, identified climate change as the environmental problem or hazard that poses *the greatest health risk* to their nation. About half of the American survey respondents were unwilling to venture even a general guess (e.g., hundreds, thousands, millions) as to how many people are being—or will in the future be—harmed worldwide by global warming. A World Health Organization study estimated that by the year 2000 climate change was causing 150,000 deaths across the globe annually, with another 5 million ‘disability-adjusted life years’ per year due to increased illness and malnutrition [25]. Of those in the United States who did guess how many deaths currently are caused by global warming, the majority underestimated by at least a factor of 10, choosing “hundreds” or “none” instead of thousands. Only 5% of Americans said correctly that estimates of current global warming injuries and illnesses are in the millions. These numbers have been publicized by the World Health Organization and in some media reports [26,27]. Yet even in Malta, when asked “What comes to your mind when you hear the terms ‘climate change’ or ‘global warming’?”, only 9.5% of respondents unprompted associated climate change with human health, and even so may be the result of confusion between greenhouse gas impacts and other types of air pollution [20].

The low salience of the human health implications of climate change should not come as a surprise. Climate change receives relatively little news coverage [28], and when it does, the human health consequences are rarely mentioned [29]. Rather, news representations and entertainment programming representations of climate change impacts tend to focus on attributes of the environment such as polar ice and glaciers, and non-human species such as polar bears and pine trees [30]. Moreover, until relatively recently, public health officials have been largely silent about climate change as a health risk [31,32]. Much of the recent public health communication activity about climate change appears to be targeted internally—from leaders in the public health community to members of the public health community at large—rather than aimed at the public. Health Canada recently conducted a study of provincial and local health authority websites and found that while 69% provide information related to health conditions that may be exacerbated by climate change, only 10% mention climate change specifically [18]. The same appears to be true of American and Maltese public health websites, although formal assessments have not been conducted.

### RQ2: Whose health does the public think will be harmed?

There were substantial differences among the three surveys with regard to questions used to assess perceptions of who is most susceptible to harm. The U.S. and Maltese questionnaires asked about harm to people both at home and abroad, while the Canadian questionnaire focused exclusively on Canadian communities and people. Furthermore, the U.S. questionnaire asked about harm to “future generations of people,” which proved to be the category of people that Americans were most likely to see as being harmed by global warming. Similarly, the Maltese questionnaire asked respondents to indicate whether they thought climate change impacts are occurring or will happen in the future.

Although the range of responses across the surveys was sizable, about a third or more of people in the United States and Canada saw themselves (United States, 32%; Canada, 67%), their family (United States, 35%; Canada, 46%), and people in their community (United States, 39%; Canada, 76%) as being vulnerable to at least moderate harm from with climate change. About one third of Maltese (31%) said they were most concerned about the risk to themselves and their families. Americans were the least likely to see themselves, their family and their community as being at risk, and viewed distant people elsewhere (in the United States, in other countries, and in future generations) as more likely to be harmed. Canadians, when asked this in an open-ended question, were most likely to see the elderly and children as most susceptible to harm; relatively few respondents pointed to other at-risk groups such as people with low incomes or who live in the Arctic or coastal regions that will be more heavily affected by climate change.

A large literature in the field of health and risk communication points to an individual’s *personal* sense of risk as the most powerful motivator of behavioral change [33,34]. This theory suggests that the closer to home a threat is, the more likely individuals will be to recognize and act on it. This may be particularly relevant in encouraging public adoption of adaptation measures to avoid increased climate health risks. A competing literature in political science—of perhaps more relevance to campaigns that seek to use public health as a frame for motivating reductions in national greenhouse gas emissions—finds that perceptions of *national* threat are sufficient drivers of policy support [35], and that the importance of self-interest in motivating behavior is over-estimated [36]. Thus, the importance of perceived personal as opposed to national health consequences of climate change is an important research question remaining to be answered.

The fact that substantial numbers of people in all countries did not view themselves as vulnerable or did not identify at-risk groups identified by scientists to be vulnerable should also not come as a surprise. Public health authorities have only just begun over the last decade to formulate approaches to identify and assess vulnerabilities in specific communities and regions [37,38] with assessments being conducted by many countries as a function of their commitment to the United Nations Framework Convention on Climate Change, including Canada [39] and Malta. Because these assessments are only relatively recent, there has been little communication of this information to the public.

### RQ3: In what specific ways does the public believe climate change will harm human health?

Canadian’s responses to an open-ended question about the ways in which climate change can harm the health of Canadians further reinforce our previously stated conclusion that the human health implications of climate change may lack salience. While 60% of Canadian respondents were able, unprompted, to name at least one specific health threat, a wide range of threats were mentioned but even the most commonly mentioned—respiratory diseases—was named by only 22% of respondents.

When specifically prompted, however, Canadians and Maltese in large numbers expressed their belief that climate change can cause respiratory/breathing problems (78–91%), heat-related problems (75–84%), cancer (61–90%), and infectious diseases (49–62%). Large numbers of Canadians also indicated sunburn (79%) and injuries from extreme weather events (73%), and large numbers of Maltese indicated allergies (84%). Some of these beliefs are misperceptions—both sunburn and (skin) cancer are likely tied to the common misperception that climate change is caused by the hole in the earth’s ozone layer [40]—but these findings indicate that the Canadian and Maltese public accept the claim that climate change can harm human health in specific ways, even if they are not based upon an accurate scientific understanding. Future educational efforts may need to focus on increasing knowledge of these specific risks and ensuring that the public is aware of their hazards, symptoms and of preventative measures.

### What actions, if any, do these findings suggest for public health officials?

Across all three countries, large numbers of people are already willing to accept that climate change has implications for human health. It is widely recognized by public health officials [8] and research scientists from other disciplines [1,2] that people in all nations need to take actions to reduce greenhouse gas emissions and to adapt to the risks posed by climate change. The willingness of Americans, Canadians and the Maltese to accept climate change as a health issue may indicate an opportunity for public health officials to educate the public not just about climate change’s health risks, but about actions needed to limit climate change and to adapt successfully to its risks.

Krosnick and colleagues [41] demonstrated that Americans who view climate change as being harmful to people are significantly more likely to support climate policy responses. More recently, research has shown that segments of the American public who understand that climate change is harming people here (rather than only in nations far away) and now (rather than at some time in the future, if at all), are more engaged in personal actions and more supportive of climate change policies [42,43]. Other studies that have assessed population behavior changes for climate change and air quality, however, have found that regional and perceptual barriers may exist as well [44,45].

Framing is an important process by which communicators can enhance their impact by linking messages and recommendations to their audience members’ deeply held values and beliefs. By defining or “framing” the relevance of climate change in ways that connect to the core values of specific audience segments—and repeatedly reinforcing that information through a variety of trusted sources and networks of recruitment—purposive communication can foster enhanced public engagement with the issue.

The public health frame—*i.e.*, that climate change is a major threat to people’s health and well-being—has considerable potential to motivate individuals to reduce greenhouse gas emissions and take adaptive actions to reduce their health risks from expected impacts. The health frame connects a complex and poorly understood topic (such as climate change) to risks the public already understand and accept as important (e.g., asthma, respiratory problems, vulnerability to extreme heat, food-borne illness and infectious disease) [30,46,47]. Several of the authors have argued that a public health frame could shift the climate debate in the United States from one based on environmental values to public health values, which are more widely held, cutting across ideology and partisanship [30,31,48]. It would also enable a new and highly respected group of voices—such as doctors, nurses and public health officials—to engage the public in the issue. And finally, it moves the location of impacts closer to home, replacing polar bears with vulnerable people, such as children, the elderly and the poor. These three surveys indicate that people in the United States, Canada and Malta are receptive to the idea that climate change will have human health impacts—and thus this may be an indication that this type of message framing is likely to be effective. People who perceive climate change as a human health threat may be more willing to adopt lifestyles that are lower in greenhouse gas emissions and support mitigation and adaptation policies. At the same time, the surveys revealed that climate change health risks are not necessarily well-known or understood, suggesting that campaigns that impart this knowledge will be viewed as imparting novel and potentially useful information.

Recent research in the United States has found that when global warming is introduced as a health problem and information is provided about how specific mitigation-related policy actions will lead to health benefits such as cleaner air to breath, healthier food to eat, and more pedestrian- and bicycle-friendly communities, a broad cross-section of Americans responded positively to this re-framing of the issue [48].

Many of the policy options to reduce greenhouse gas emissions provide direct societal benefits from improved public health, thereby offsetting some of the often more apparent costs of carbon tax, cap or regulatory mechanisms [46]. Public health officials can assist policymakers responsible for actions to reduce greenhouse gas emissions by heightening their awareness of the health co-benefits of climate policies and their monetary value. Recently, a series of papers in the Lancet quantified health outcomes from increased household energy efficiency, walking and cycling, less consumption of animal products, and cleaner fuels and technologies in order to better integrate health gains, and cost savings, into climate policy decisions [47,49–53]. A public health approach to climate change may also have more relevance at local governmental levels. More walkable communities, public transit systems and urban reforestation serve to protect global climate [46], but of perhaps more relevance to local officials, they also directly reduce air pollution levels in their municipalities, and may aid them in achieving other environmental objectives, such as reduced ground level ozone.

In Canada, health promotion programs already exist that attempt to motivate individuals to reduce their personal risks from climate-related hazards such as West Nile virus, smog, extreme heat and food safety [18]; similar programs exist in the United States and Malta. The 2009 Health Canada report found that virtually all materials produced by these programs on climate-related risks do not refer to climate change, and moreover, even with dissemination of the materials, many Canadians still are not adopting health-protective behaviors. There is little evidence on whether the use of a climate change public health frame in engaging the public on adaptation to these risks would be more effective, however. This raises an important issue that should be addressed in additional research.

### What, if any, additional research should be undertaken?

Little social science research exists to date on the ways in which people—in any country—are thinking about the health risks from climate change. As posited above, introducing a new frame for people to use in understanding the complex issue of climate change may serve to bring a new dimension to help efforts of public health officials increase public knowledge of climate change health risks and motivate individuals to take adaptive and greenhouse gas reduction actions. Several of the authors on this paper are currently conducting research on the effectiveness of a public health message frame compared to traditional environmental and national security frames, and are analyzing the differences in message appeal across audience segments that have previously been defined by their attitudes, beliefs, actions and policy preferences on global warming [42,43]. We expect that the manner in which various audiences process public health information is likely to be influenced by their deeply held values, attitudes and beliefs, and is also a function of variables that differ across individuals, such as political ideology [54] or socioeconomics, as well as those that vary at broader scales, such as national cultural traits [24]. The development of uniform measures of climate health beliefs, risk perceptions and adaptation actions will provide a yardstick by which comparisons can be more easily made at all levels and by teams of researchers working independently, with the end goal of the development of more effective public health outreach campaigns on climate change at all levels—local, regional, national and international.

However useful surveys may be in broadly understanding the public’s perceptions, more fine-grained information about the mental models they use in processing this information will also need to be obtained using techniques such as those established by Baruch Fischhoff at Carnegie Mellon [55]. In-depth interviews with both members of the scientific community and the public on climate change health risks and adaptation responses will be needed in order to learn what types of information will be most valuable to audiences in affecting behavioral changes to reduce their risks from climate change as individuals and communities. Surveys, such as conducted in Malta, that obtain richer narrative data may also provide a window to greater understanding of the ways that the public intersects with this issue.

## 5. Conclusion

The public health community has an opportunity to frame the issue of climate change in a manner that promotes the engagement of individuals, governments, and a range of other stakeholders. Doing so will likely build support for policies that will mitigate climate change and help communities successfully adapt to unavoidable changes, and will encourage individuals to take actions to reduce their own contributions to climate change and protect themselves from its impacts. In the face of aggressive counter-claims against climate science, public beliefs in and concerns about climate change have recently declined in the United States [56] and Europe [57]. This opens a window of opportunity for the public health community to draw attention to climate change’s human health consequences using a communication strategy that has proven effective in ameliorating a range of public health problems: simple clear messages, repeated often, by a variety of trusted public health voices within a wider policy environment that supports greenhouse gas reduction behavior and healthy lifestyles.

## Figures and Tables

**Figure S1.**
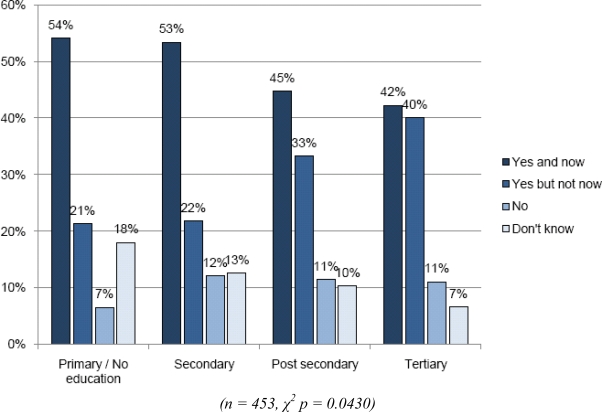
Maltese perceptions of risk of death from climate change by educational level.

## Appendix

**Table S1.** National sample demographic characteristics.

	**U.S.**	**Canada**	**Malta**
**Gender**			
Male	48%	48%	49%
Female	52%	52%	51%
**Age group**			
18–24 yrs	11%	11%	12%
25–34 yrs	19%	16%	16%
35–44 yrs	19%	20%	16%
45–54 yrs	18%	20%	21%
55–64 yrs	18%	15%	18%
65–74 yrs	11%	65+ yrs: 18%	11%
75+ yrs	5%		6%
**Education level**			
	Less than high school: 13%		Primary: 17%
	High school: 32%	High school or less: 25%	Secondary: 48%
	Some college: 28%	College: 28%	Post-secondary: 18%
	Bachelor’s degree or higher: 27%	University +: 46%	Tertiary: 17%

**Table S2.** U.S. perceptions of global warming impacts by educational level; *Worldwide over the next 20 years, do you think global warming will cause more or less of the following, if nothing is done to address it?*

**%**	**National average**	Less than high school	High school	Some college	Bachelor's degree or higher
***Droughts and water shortages***					
*Many more*	**43**	50	38	42	46
*A few more*	**22**	14	24	20	25
*No difference*	**14**	7	13	15	16
*A few less*	**1**	3	1	1	1
*Many less*	**1**	1	1	2	0
*Don’t know*	**19**	25	23	20	11
*χ2, p < 0.01; n = 2,129*					
***People living in poverty***					
*Many more*	**33**	34	29	34	38
*A few more*	**18**	19	21	16	18
*No difference*	**22**	12	21	24	27
*A few less*	**0**	1	1	0	0
*Many less*	**1**	1	0	2	1
*Don’t know*	**25**	32	28	25	17
*χ2, p < 0.01; n = 2,137*					
***Disease epidemics***					
*Many more*	**31**	40	28	31	31
*A few more*	**22**	13	23	22	23
*No difference*	**20**	9	18	21	25
*A few less*	**1**	1	1	0	1
*Many less*	**1**	0	0	1	1
*Don’t know*	**26**	38	29	25	19
*χ2, p < 0.01; n = 2,131*					
***Intense hurricanes***					
*Many more*	**39**	38	36	40	42
*A few more*	**23**	16	24	23	23
*No difference*	**15**	8	14	15	20
*A few less*	**0**	1	1	0	1
*Many less*	**1**	0	1	2	1
*Don’t know*	**22**	37	25	20	14
*χ2, p < 0.01; n = 2,141*					
***Intense rainstorms***					
*Many more*	**39**	42	35	39	42
*A few more*	**24**	19	27	22	25
*No difference*	**15**	8	14	16	19
*A few less*	**1**	1	2	0	0
*Many less*	**1**	0	0	2	1
*Don’t know*	**20**	31	22	21	12
*χ2, p<0.01; n = 2,130*					
***Severe heat waves***					
*Many more*	**42**	43	37	42	45
*A few more*	**24**	18	26	24	26
*No difference*	**14**	8	13	14	18
*A few less*	**1**	1	1	0	1
*Many less*	**1**	0	0	2	0
*Don’t know*	**19**	30	22	17	10
*χ2, p<0.01; n = 2,136*					
***Famines and food shortages***					
*Many more*	**39**	44	34	38	43
*A few more*	**24**	14	25	24	27
*No difference*	**15**	9	14	16	17
*A few less*	**1**	2	1	0	1
*Many less*	**1**	1	1	2	0
*Don’t know*	**21**	30	25	20	12
*χ2, p < 0.01; n = 2,140*					

**Table S3.** U.S. perceptions of global warming impacts by income level; *Worldwide over the next 20 years, do you think global warming will cause more or less of the following, if nothing is done to address it?*

**%**	**National average**	Less than $25,000	$25,000 to $34,999	$35,000 to $49,999	$50,000 to $74,999	$75,000 to $99,999	$100,000 or more
***Droughts and water shortages***							
*Many more*	**43**	49	44	41	42	40	42
*A few more*	**22**	15	22	21	24	28	25
*No difference*	**14**	9	12	13	15	16	19
*A few less*	**1**	3	0	2	1	2	1
*Many less*	**1**	1	3	0	1	1	0
*Don’t know*	**19**	24	18	23	17	13	13
*χ2, p < 0.01; n = 2,112*							
***People living in poverty***							
*Many more*	**33**	35	35	33	34	30	32
*A few more*	**19**	18	20	15	17	20	23
*No difference*	**23**	16	18	22	24	31	26
*A few less*	**0**	1	0	1	0	0	0
*Many less*	**1**	0	3	1	1	1	0
*Don’t know*	**24**	31	23	28	23	19	18
*χ2, p < 0.01; n = 2,120*							
***Disease epidemics***							
*Many more*	**31**	35	34	34	29	27	29
*A few more*	**22**	21	20	19	24	22	25
*No difference*	**20**	12	18	17	21	28	25
*A few less*	**1**	1	1	1	1	0	1
*Many less*	**1**	0	0	0	1	1	0
*Don’t know*	**26**	31	26	30	24	22	20
*χ2, p < 0.01; n = 2,117*							
***Intense hurricanes***							
*Many more*	**39**	44	37	39	40	38	36
*A few more*	**23**	17	20	23	26	27	25
*No difference*	**15**	11	14	14	13	17	24
*A few less*	**0**	1	0	0	0	1	1
*Many less*	**1**	0	3	0	1	1	0
*Don’t know*	**21**	27	26	24	20	17	14
*χ2, p < 0.01; n = 2,123*							
***Intense rainstorms***							
*Many more*	**39**	45	37	39	39	34	38
*A few more*	**24**	17	25	24	25	31	25
*No difference*	**15**	10	13	14	15	18	22
*A few less*	**1**	1	0	0	1	1	0
*Many less*	**1**	0	3	0	1	1	0
*Don’t know*	**20**	26	22	22	19	15	14
*χ2, p < 0.01; n = 2,114*							
***Severe heat waves***							
*Many more*	**42**	47	39	40	42	41	39
*A few more*	**25**	17	28	23	27	27	28
*No difference*	**14**	10	14	12	11	19	21
*A few less*	**1**	2	0	0	1	0	1
*Many less*	**1**	0	3	0	1	1	0
*Don’t know*	**18**	23	17	24	18	12	11
*χ2, p < 0.01; n = 2,125*							
***Famines and food shortages***							
*Many more*	**39**	43	42	37	39	36	39
*A few more*	**24**	17	21	26	26	25	28
*No difference*	**15**	10	15	12	15	20	18
*A few less*	**1**	2	0	1	0	1	1
*Many less*	**1**	0	3	1	1	1	0
*Don’t know*	**20**	28	18	23	19	17	14
*χ2, p < 0.01; n = 2,125*							

**Table S4.** U.S. perceptions of global warming impacts by age category; *Worldwide over the next 20 years, do you think global warming will cause more or less of the following, if nothing is done to address it?*

**%**	**National average**	18–29	30–44	45–59	60+
***Droughts and water shortages***					
*Many more*	**43**	40	43	47	42
*A few more*	**22**	22	24	20	22
*No difference*	**14**	16	14	12	14
*A few less*	**1**	1	1	2	2
*Many less*	**1**	2	0	1	1
*Don’t know*	**19**	20	18	19	20
*χ2, p = 0.130; n = 2,128*					
***People living in poverty***					
*Many more*	**33**	27	30	42	32
*A few more*	**18**	20	21	15	18
*No difference*	**22**	28	22	18	23
*A few less*	**0**	1	1	0	0
*Many less*	**1**	2	0	1	0
*Don’t know*	**25**	23	26	23	27
*χ2, p < 0.01; n = 2,133*					
***Disease epidemics***					
*Many more*	**31**	28	29	34	32
*A few more*	**22**	26	22	20	20
*No difference*	**20**	21	22	17	19
*A few less*	**1**	0	1	1	0
*Many less*	**1**	0	0	1	1
*Don’t know*	**26**	25	25	27	29
*χ2, p = 0.098; n = 2,135*					
***Intense hurricanes***					
*Many more*	**39**	35	40	43	37
*A few more*	**23**	25	26	19	21
*No difference*	**15**	15	14	15	16
*A few less*	**0**	0	1	0	0
*Many less*	**1**	2	1	1	0
*Don’t know*	**22**	23	19	21	25
*χ2, p < 0.028; n = 2,138*					
***Intense rainstorms***					
*Many more*	**39**	35	38	44	38
*A few more*	**24**	26	24	22	25
*No difference*	**15**	16	16	14	15
*A few less*	**1**	1	2	0	0
*Many less*	**1**	2	1	1	0
*Don’t know*	**20**	20	20	20	21
*χ2, p < 0.061; n = 2,133*					
***Severe heat waves***					
*Many more*	**42**	39	44	45	38
*A few more*	**24**	23	27	21	26
*No difference*	**14**	15	13	14	13
*A few less*	**1**	1	1	1	1
*Many less*	**1**	2	0	1	1
*Don’t know*	**19**	21	16	18	21
*χ2, p < 0.186; n = 2,140*					
***Famines and food shortages***					
*Many more*	**39**	32	38	44	39
*A few more*	**24**	26	27	21	21
*No difference*	**15**	17	15	13	15
*A few less*	**1**	1	1	1	1
*Many less*	**1**	2	1	2	1
*Don’t know*	**21**	23	19	20	22
*χ2, p < 0.034; n = 2,139*					

**Table S5.** U.S. perceptions of timing of harm to people in the United States by educational level; *When do you think global warming will start to harm people in the United States?*

	**National average**	Less than high school	High school	Some college	Bachelor's degree or higher
*They are being harmed now*	**34**	32	35	34	34
*In 10 years*	**13**	15	14	10	14
*In 25 years*	**13**	15	13	12	13
*In 50 years*	**13**	14	15	13	11
*In 100 years*	**12**	11	11	14	11
*Never*	**15**	13	12	17	16
*χ2, p = 0.258; n = 2,097*					

**Table S6.** U.S. perceptions of timing of harm to people in the United States by income level; *When do you think global warming will start to harm people in the United States?*

	**National average**	Less than $25,000	$25,000 to $34,999	$35,000 to $49,999	$50,000 to $74,999	$75,000 to $99,999	$100,000 or more
*They are being harmed now*	**34**	40	43	30	35	28	28
*In 10 years*	**13**	14	12	13	11	12	17
*In 25 years*	**13**	12	12	10	19	13	11
*In 50 years*	**13**	13	13	16	13	11	14
*In 100 years*	**12**	9	7	15	9	18	11
*Never*	**15**	11	12	17	13	18	20
*χ2, p = 0.258; n = 2,082*							

**Table S7.** U.S. perceptions of timing of harm to people in the United States by age; *When do you think global warming will start to harm people in the United States?*

	**National average**	18–29	30–44	45–59	60+
*They are being harmed now*	**34**	30	30	41	33
*In 10 years*	**13**	12	16	11	14
*In 25 years*	**13**	17	11	12	13
*In 50 years*	**13**	14	15	10	15
*In 100 years*	**12**	13	14	11	10
*Never*	**15**	14	13	16	15
*χ2, p < 0.01; n = 2,097*					

**Table S8.** U.S. perceptions of timing of harm to people worldwide by educational level; *When do you think global warming will start to harm other people around the world?*

	**National average**	Less than high school	High school	Some college	Bachelor's degree or higher
*They are being harmed now*	**38**	35	41	36	39
*In 10 years*	**13**	15	11	12	13
*In 25 years*	**12**	14	12	12	12
*In 50 years*	**12**	12	13	12	11
*In 100 years*	**11**	9	12	12	10
*Never*	**14**	15	12	16	15
*χ2, p = 0.513; n = 2,090*					

**Table S9.** U.S. perceptions of timing of harm to people worldwide by income level; *When do you think global warming will start to harm other people around the world?*

	**National average**	Less than $25,000	$25,000 to $34,999	$35,000 to $49,999	$50,000 to $74,999	$75,000 to $99,999	$100,000 or more
*They are being harmed now*	**38**	46	45	34	38	32	33
*In 10 years*	**13**	10	13	12	13	13	16
*In 25 years*	**12**	12	12	10	16	12	10
*In 50 years*	**12**	10	11	14	12	12	12
*In 100 years*	**11**	9	7	14	9	14	12
*Never*	**14**	12	11	16	11	17	17
*χ2, p < 0.01; n = 2,073*							

**Table S10.** U.S. perceptions of timing of harm to people worldwide by age category; *When do you think global warming will start to harm other people around the world?*

	**National average**	18–29	30–44	45–59	60+
*They are being harmed now*	**38**	30	37	45	39
*In 10 years*	**13**	13	15	10	12
*In 25 years*	**12**	18	9	11	13
*In 50 years*	**12**	14	14	8	13
*In 100 years*	**11**	11	13	11	9
*Never*	**14**	13	13	15	14
*χ2, p < 0.01; n = 2,091*					

**Table S11.** U.S. perceptions of morbidity and mortality rates by educational level; *Now please think about the human health effects of global warming. (Please choose the answer corresponding to your best estimate.) Worldwide, how many people do you think?*

**%**	**National average**	Less than high school	High school	Some college	Bachelor's degree or higher
***Currently die each year due to global warming?***					
*Millions*	**3**	3	3	3	4
*Thousands*	**14**	12	12	11	19
*Hundreds*	**12**	13	9	14	12
*None*	**23**	20	20	24	27
*Don't Know*	**48**	52	56	48	38
*χ2, p<0.01; n = 2,138*					
***Will die each year 50 years from now due to global warming?***					
*Millions*	**11**	10	6	13	15
*Thousands*	**17**	14	16	14	22
*Hundreds*	**8**	6	9	8	8
*None*	**14**	15	11	16	15
*Don't Know*	**50**	56	57	49	40
*χ2, p<0.01; n = 2,147*					
***Are currently injured or become ill each year due to global warming?***					
*Millions*	**5**	6	3	5	5
*Thousands*	**15**	15	13	14	19
*Hundreds*	**13**	10	11	15	15
*None*	**21**	21	17	23	24
*Don't Know*	**46**	48	56	43	36
*χ2, p < 0.01; n = 2,143*					
***Will be injured or become ill each year 50 years from now due to global warming?***					
*Millions*	**13**	12	9	13	17
*Thousands*	**15**	13	14	14	19
*Hundreds*	**7**	8	8	7	8
*None*	**15**	14	12	17	15
*Don't Know*	**50**	53	57	49	41
*χ2, p<0.01; n = 2,124*					

**Table S12.** U.S. perceptions of morbidity and mortality rates by income level; *Now please think about the human health effects of global warming. (Please choose the answer corresponding to your best estimate.) Worldwide, how many people do you think?*

**%**	**National average**	Less than $25,000	$25,000 to $34,999	$35,000 to $49,999	$50,000 to $74,999	$75,000 to $99,999	$100,000 or more
***Currently die each year due to global warming?***							
*Millions*	**3**	4	1	5	3	2	4
*Thousands*	**14**	17	15	11	13	12	17
*Hundreds*	**12**	8	10	9	14	15	8
*None*	**23**	17	22	24	23	30	17
*Don't Know*	**48**	55	52	51	47	41	55
*χ2, p<0.01; n = 2,120*							
***Will die each year 50 years from now due to global warming?***							
*Millions*	**11**	11	7	11	12	10	14
*Thousands*	**17**	16	19	12	15	20	21
*Hundreds*	**8**	7	4	10	12	6	7
*None*	**15**	9	14	15	15	18	19
*Don't Know*	**50**	57	55	53	46	46	39
*χ2, p < 0.01; n = 2,125*							
***Are currently injured or become ill each year due to global warming?***							
*Millions*	**5**	5	4	5	4	4	5
*Thousands*	**15**	17	16	14	14	13	16
*Hundreds*	**13**	9	9	12	15	16	15
*None*	**22**	14	20	21	21	28	26
*Don't Know*	**46**	54	51	48	45	38	38
*χ2, p < 0.01; n = 2,120*							
***Will be injured or become ill each year 50 years from now due to global warming?***							
*Millions*	**13**	14	8	10	13	15	16
*Thousands*	**15**	14	20	13	13	15	19
*Hundreds*	**8**	7	3	8	11	7	6
*None*	**15**	9	14	16	15	17	19
*Don't Know*	**50**	56	55	54	47	46	40
*χ2, p < 0.01; n = 2,103*							

**Table S13.** U.S. perceptions of morbidity and mortality rates by age; *Now please think about the human health effects of global warming. (Please choose the answer corresponding to your best estimate.) Worldwide, how many people do you think?*

**%**	**National average**	18–29	30–44	45–59	60+
***Currently die each year due to global warming?***					
*Millions*	**3**	2	2	4	3
*Thousands*	**14**	12	13	17	12
*Hundreds*	**12**	15	13	10	9
*None*	**23**	28	22	21	22
*Don't Know*	**48**	42	50	48	53
*χ2, p = 0.001; n = 2,141*					
***Will die each year 50 years from now due to global warming?***					
*Millions*	**11**	11	10	14	8
*Thousands*	**17**	17	21	14	14
*Hundreds*	**8**	11	8	8	7
*None*	**14**	16	14	15	12
*Don't Know*	**50**	45	47	49	59
*χ2, p < 0.01; n = 2,147*					
***Are currently injured or become ill each year due to global warming?***					
*Millions*	**5**	4	4	7	4
*Thousands*	**15**	16	15	17	13
*Hundreds*	**13**	18	13	12	9
*None*	**21**	24	22	20	21
*Don't Know*	**46**	38	47	45	54
*χ2, p < 0.01; n = 2,120*					
***Will be injured or become ill each year 50 years from now due to global warming?***					
*Millions*	**13**	12	13	16	10
*Thousands*	**15**	17	17	14	13
*Hundreds*	**7**	9	8	7	6
*None*	**15**	17	14	15	14
*Don't Know*	**50**	46	48	48	57
*χ2, p < 0.01; n = 2,103*					

**Table S14.** Canadian perceptions of environmental impacts resulting from climate change by age; *I will now read you a list of potential risks to the health of Canadians. Please tell me whether you think each of the following poses a major risk, a moderate risk, a minor risk, or no risk at all to the health of Canadians. [Read only 8 of 12 items to reduce burden response] (n = 1,600).*

	**National average**	18–34	35–49	50–64	65+
Obesity (n = 938)	**70**	61	74	73	71
Heart disease (n = 1,146)	**65**	59	70	72	73
Air pollution (n = 1,154)	**62**	59	64	65	62
Chemical pollution (n = 1,145)	**58**	46	64	60	60
Second-hand smoke (n = 1,139)	**57**	56	54	58	65
Pesticides in food (n = 919)	**46**	32	50	53	52
Climate change (n = 902)	**32**	**35**	**32**	**30**	**28**
Pandemic flu epidemics (n = 1,159)	**29**	23	26	34	39
Heat waves (n = 916)	**20**	18	16	23	26
West Nile virus (n = 1,168)	**16**	13	15	16	22
Extreme cold weather (n = 1,165)	**15**	14	10	17	19
Tap water (n = 903)	**14**	15	14	13	15

**Table S15.** Maltese perceptions of likelihood of health risks resulting from climate change by age; *How likely do you think it is that each of the following will occur during the next 50 years due to climate change? [Index: 1 = very unlikely to 4 = very likely]*

	**National average**	18–34	35–54	55+
Worldwide, many people’s standard of living will decrease due to climate change.	**2.85**	2.82	2.94	2.76
Worldwide, water shortages will occur due to climate change.	**2.85**	2.82	2.99	2.73
Increased rates of serious disease worldwide due to climate change.	**3.28**	3.24	3.36	3.22
You or your family’s standard of living will decrease due to climate change.	**2.65**	2.57	2.73	2.64
Water shortages will occur in Malta due to climate change.	**2.66**	2.52	2.75	2.67
The chance of you or your family getting a serious disease will increase due to climate change.	**3.00**	2.96	2.99	3.05

**Table S16.** Maltese perceptions of likelihood of health risks resulting from climate change by educational level; *How likely do you think it is that each of the following will occur during the next 50 years due to climate change? [Index: 1 = very unlikely to 4 = very likely]*

	**National average**	Primary / No education	Secondary	Post secondary	Tertiary
Worldwide, many people’s standard of living will decrease due to climate change.	**2.85**	2.75	2.93	2.83	2.72
Worldwide, water shortages will occur due to climate change.	**2.85**	2.51	2.85	2.90	3.04
Increased rates of serious disease worldwide due to climate change.	**3.28**	3.25	3.33	3.21	3.26
You or your family’s standard of living will decrease due to climate change.	**2.65**	2.57	2.76	2.65	2.47
Water shortages will occur in Malta due to climate change.	**2.66**	2.60	2.73	2.54	2.64
The chance of you or your family getting a serious disease will increase due to climate change.	**3.00**	3.16	3.05	2.85	2.92

**Table S17.** U.S. perceptions of how much people will be harmed by educational level; *How much do you think global warming will harm?*

	**National average**	Less than high school	High school	Some college	Bachelor's degree or higher
***You personally***					
*A great deal*	**10**	19	9	8	7
*A moderate amount*	**22**	18	20	22	25
*Only a little*	**24**	18	20	27	29
*Not at all*	**22**	17	21	23	24
*Don't know*	**23**	29	31	20	15
*A great deal*	**10**	19	9	8	7
*Don't know*	**22**	18	20	22	25
*χ2, p < 0.01; n = 2,139*					
***Your family***					
*A great deal*	**11**	18	10	10	10
*A moderate amount*	**24**	19	24	25	26
*Only a little*	**23**	21	19	24	27
*Not at all*	**19**	14	16	21	21
*Don't know*	**23**	28	31	20	15
*χ2, p < 0.01; n = 2,135*					
***Your community***					
*A great deal*	**13**	20	12	13	12
*A moderate amount*	**26**	21	26	25	30
*Only a little*	**20**	14	18	23	23
*Not at all*	**17**	13	14	19	20
*Don't know*	**23**	32	30	20	15
*χ2, p < 0.01; n = 2,137*					
***People in the United States***					
*A great deal*	**21**	28	20	20	21
*A moderate amount*	**28**	21	26	29	33
*Only a little*	**15**	9	15	17	16
*Not at all*	**14**	11	12	15	16
*Don't know*	**22**	31	28	19	14
*χ2, p < 0.01; n = 2,145*					
***People in other modern industrialized countries***					
*A great deal*	**22**	25	21	22	23
*A moderate amount*	**28**	19	27	27	33
*Only a little*	**13**	9	12	15	15
*Not at all*	**13**	9	11	15	15
*Don't know*	**24**	37	29	22	15
*χ2, p < 0.01; n = 2,137*					
***People in developing countries***					
*A great deal*	**31**	31	24	32	40
*A moderate amount*	**22**	17	26	19	21
*Only a little*	**10**	6	11	13	10
*Not at all*	**13**	10	10	15	15
*Don't know*	**24**	36	30	21	14
*χ2, p < 0.01; n = 2,134*					
***Future generations of people***					
*A great deal*	**44**	45	36	45	51
*A moderate amount*	**17**	12	20	16	16
*Only a little*	**7**	6	8	7	7
*Not at all*	**10**	8	7	11	13
*Don't know*	**22**	28	28	21	13
*χ2, p < 0.01; n = 2,130*					

**Table S18.** U.S. perceptions of how much people will be harmed by income level; *How much do you think global warming will harm?*

	**National average**	Less than $25,000	$25,000 to $34,999	$35,000 to $49,999	$50,000 to $74,999	$75,000 to $99,999	$100,000 or more
***You personally***							
*A great deal*	**10**	13	6	12	8	9	8
*A moderate amount*	**22**	19	24	22	22	21	24
*Only a little*	**24**	20	20	20	25	31	29
*Not at all*	**22**	15	24	24	22	26	24
*Don't know*	**23**	34	25	23	22	13	14
*χ2, p < 0.01; n = 2,123*							
***Your family***							
*A great deal*	**11**	14	8	13	11	8	11
*A moderate amount*	**24**	20	29	22	26	27	24
*Only a little*	**23**	21	17	22	24	27	27
*Not at all*	**19**	10	22	20	18	25	22
*Don't know*	**23**	35	24	23	22	13	15
*χ2, p < 0.01; n = 2,121*							
***Your community***							
*A great deal*	**13**	17	11	13	13	9	14
*A moderate amount*	**26**	24	30	25	27	27	25
*Only a little*	**21**	18	14	18	22	28	24
*Not at all*	**17**	8	19	18	17	22	21
*Don't know*	**23**	33	25	26	21	14	15
*χ2, p < 0.01; n = 2,124*							
***People in the United States***							
*A great deal*	**22**	28	22	20	20	20	18
*A moderate amount*	**28**	25	26	28	31	28	30
*Only a little*	**15**	10	11	14	17	21	19
*Not at all*	**14**	7	18	14	13	18	17
*Don't know*	**22**	30	24	25	19	13	16
*χ2, p < 0.01; n = 2,128*							
***People in other modern industrialized countries***							
*A great deal*	**22**	28	21	22	21	23	18
*A moderate amount*	**27**	23	25	25	32	28	32
*Only a little*	**13**	11	11	12	13	18	17
*Not at all*	**13**	6	16	13	13	17	17
*Don't know*	**24**	32	27	28	21	15	17
*χ2, p < 0.01; n = 2,126*							
***People in developing countries***							
*A great deal*	**32**	33	28	29	29	34	37
*A moderate amount*	**22**	21	18	20	24	23	21
*Only a little*	**11**	7	10	11	13	13	10
*Not at all*	**13**	7	16	13	13	16	16
*Don't know*	**24**	32	27	27	22	14	16
*χ2, p < 0.01; n = 2,123*							
***Future generations of people***							
*A great deal*	**44**	44	41	38	42	50	51
*A moderate amount*	**17**	14	18	17	22	15	14
*Only a little*	**7**	6	6	10	7	7	7
*Not at all*	**10**	5	12	11	8	13	14
*Don't know*	**22**	31	23	24	21	14	13
*χ2, p < 0.01; n = 2,111*							

**Table S19.** U.S. perceptions of how much people will be harmed by age category; *How much do you think global warming will harm?*

	**National average**	18–29	30–44	45–59	60+
***You personally***					
*A great deal*	**10**	8	13	11	6
*A moderate amount*	**22**	23	21	23	21
*Only a little*	**24**	27	26	22	21
*Not at all*	**22**	25	20	19	23
*Don't know*	**23**	18	20	25	27
*χ2, p < 0.01; n = 2,144*					
***Your family***					
*A great deal*	**11**	8	13	13	9
*A moderate amount*	**24**	25	22	25	25
*Only a little*	**23**	27	25	20	21
*Not at all*	**19**	21	19	17	18
*Don't know*	**23**	19	20	25	28
*χ2, p = 0.001; n = 2,139*					
***Your community***					
*A great deal*	**13**	12	14	16	10
*A moderate amount*	**26**	25	25	26	28
*Only a little*	**20**	24	23	17	19
*Not at all*	**17**	20	16	16	16
*Don't know*	**23**	19	22	25	27
*χ2, p = 0.004; n = 2,136*					
***People in the United States***					
*A great deal*	**21**	18	19	27	20
*A moderate amount*	**28**	30	32	24	27
*Only a little*	**15**	17	14	14	15
*Not at all*	**14**	17	13	13	12
*Don't know*	**22**	18	22	22	25
*χ2, p = 0.001; n = 2,128*					
***People in other modern industrialized countries***					
*A great deal*	**22**	19	18	27	24
*A moderate amount*	**28**	30	33	23	25
*Only a little*	**13**	15	12	12	15
*Not at all*	**13**	16	13	13	10
*Don't know*	**24**	21	24	24	27
*χ2, p < 0.01; n = 2,138*					
***People in developing countries***					
*A great deal*	**31**	31	31	35	29
*A moderate amount*	**21**	23	22	19	22
*Only a little*	**11**	11	11	9	12
*Not at all*	**13**	16	12	13	10
*Don't know*	**24**	19	24	24	27
*χ2, p = 0.045; n = 2,136*					
***Future generations of people***					
*A great deal*	**44**	45	44	46	40
*A moderate amount*	**17**	16	19	15	17
*Only a little*	**7**	8	7	5	10
*Not at all*	**10**	13	8	11	7
*Don't know*	**22**	18	21	23	26
*χ2, p = 0.001; n = 2,130*					

**Table S20.** U.S. concern about who will be impacted by global warming by educational level; *How concerned are you about the impact of global warming on…. [all people, all children, your children, people in the U.S., you, your health].[not at all concerned = 1, extremely concerned =7]*

	**National average**	Less than high school	High school	Some college	Bachelor's degree or higher
***All people***					
*7*	**24**	31	25	22	22
*6*	**16**	16	15	17	17
*5*	**19**	14	18	20	20
*4*	**19**	19	23	17	16
*3*	**8**	8	7	9	7
*2*	**5**	6	5	5	6
*1*	**9**	6	8	10	11
*χ2, p = 0.016; n = 2,135*					
***All children***					
*7*	**30**	45	31	26	26
*6*	**17**	13	15	21	18
*5*	**17**	9	18	16	21
*4*	**16**	17	18	15	13
*3*	**7**	8	6	8	5
*2*	**5**	3	5	5	6
*1*	**8**	5	8	8	10
*χ2, p<0.01; n=2,129*					
***Your children***					
*7*	**30**	39	29	29	26
*6*	**15**	14	13	16	18
*5*	**15**	13	16	14	15
*4*	**15**	17	18	15	12
*3*	**6**	7	6	7	6
*2*	**5**	3	5	4	6
*1*	**14**	8	13	14	17
*χ2, p < 0.01; n = 2,109*					
***People in the U.S.***					
*7*	**21**	27	23	19	18
*6*	**16**	17	14	17	16
*5*	**19**	13	19	20	21
*4*	**21**	20	23	19	21
*3*	**8**	14	7	9	7
*2*	**6**	4	6	5	7
*1*	**9**	5	8	10	10
*χ2, p < 0.01; n = 2,141*					
***You***					
*7*	**21**	23	24	21	17
*6*	**14**	19	11	13	15
*5*	**17**	14	18	20	16
*4*	**21**	25	22	19	20
*3*	**9**	7	7	12	10
*2*	**7**	4	7	6	9
*1*	**11**	8	11	10	13
*χ2, p < 0.01; n = 2,117*					
***Your health***					
*7*	**24**	38	25	22	18
*6*	**15**	14	14	15	17
*5*	**17**	13	15	20	18
*4*	**19**	16	22	19	17
*3*	**9**	8	8	9	10
*2*	**6**	4	6	6	8
*1*	**10**	7	10	10	13
*χ2, p < 0.01; n = 2,134*					
***Your lifestyle***					
*7*	**13**	24	13	13	9
*6*	**10**	10	10	8	11
*5*	**17**	17	16	19	14
*4*	**25**	23	28	24	24
*3*	**12**	8	10	14	14
*2*	**10**	8	10	9	12
*1*	**13**	11	13	13	15
*χ2, p < 0.01; n = 2,126*					
***Your future***					
*7*	**21**	33	23	19	16
*6*	**15**	19	14	16	15
*5*	**19**	11	16	22	22
*4*	**19**	15	23	17	18
*3*	**9**	10	9	10	10
*2*	**6**	7	6	6	7
*1*	**10**	6	10	10	13
*χ2, p < 0.01; n = 2,131*					

**Table S21.** U.S. concern about who will be impacted by global warming by income level; *How concerned are you about the impact of global warming on…. [all people, all children, your children, people in the U.S., you, your health].[not at all concerned = 1, extremely concerned =7]*

	**National average**	Less than $25,000	$25,000 to $34,999	$35,000 to $49,999	$50,000 to $74,999	$75,000 to $99,999	$100,000 or more
***All people***							
*7*	**25**	34	25	21	23	21	20
*6*	**17**	19	12	18	17	17	15
*5*	**19**	14	20	18	20	23	19
*4*	**19**	17	18	20	23	17	16
*3*	**7**	6	9	9	7	6	8
*2*	**5**	3	7	4	5	8	7
*1*	**9**	7	10	10	7	9	14
*χ2, p < 0.01; n = 2,113*							
***All children***							
*7*	**30**	44	25	29	27	26	25
*6*	**17**	15	14	18	19	20	16
*5*	**17**	13	22	15	19	18	19
*4*	**16**	14	18	14	18	16	14
*3*	**7**	6	8	9	7	4	7
*2*	**5**	3	5	6	3	6	7
*1*	**8**	6	7	9	7	9	13
*χ2, p < 0.01; n = 2,113*							
***Your children***							
*7*	**30**	39	24	30	27	29	25
*6*	**15**	14	13	16	17	17	15
*5*	**15**	12	17	13	18	15	13
*4*	**15**	14	19	13	17	15	17
*3*	**7**	7	8	9	7	3	5
*2*	**5**	4	5	4	4	7	6
*1*	**13**	11	13	14	11	15	19
*χ2, p = 0.01; n = 2,095*							
***People in the U.S.***							
*7*	**21**	32	21	20	18	17	16
*6*	**16**	19	10	14	16	15	17
*5*	**19**	14	20	22	22	19	18
*4*	**21**	18	19	19	26	26	18
*3*	**8**	9	10	10	7	6	8
*2*	**6**	3	9	4	4	7	9
*1*	**9**	6	11	10	6	10	13
*χ2, p < 0.01; n = 2,119*							
***You***							
*7*	**21**	34	19	21	18	16	14
*6*	**14**	13	12	17	15	15	12
*5*	**17**	15	16	17	19	22	17
*4*	**21**	19	23	17	23	20	22
*3*	**9**	7	11	10	10	8	9
*2*	**7**	4	9	6	5	8	10
*1*	**11**	8	11	12	10	11	15
*χ2, p < 0.01; n = 2,095*							
***Your health***							
*7*	**24**	39	20	25	19	16	17
*6*	**16**	13	10	19	15	19	16
*5*	**17**	14	22	12	20	18	16
*4*	**18**	15	21	16	21	21	17
*3*	**9**	7	9	11	10	6	10
*2*	**6**	5	6	6	5	8	9
*1*	**10**	7	11	11	9	11	15
*χ2, p < 0.01; n = 2,114*							
***Your lifestyle***							
*7*	**13**	22	15	15	7	8	10
*6*	**10**	12	8	8	10	9	11
*5*	**17**	15	18	15	18	20	15
*4*	**25**	23	20	25	30	28	22
*3*	**12**	10	16	14	12	10	12
*2*	**10**	7	13	8	11	12	12
*1*	**13**	11	12	14	13	13	18
*χ2, p < 0.01; n = 2,105*							
***Your future***							
*7*	**21**	33	22	23	16	14	16
*6*	**16**	17	6	17	17	18	15
*5*	**19**	16	20	15	20	23	20
*4*	**19**	15	21	17	25	19	17
*3*	**9**	7	11	12	8	9	9
*2*	**6**	5	11	6	5	7	8
*1*	**10**	7	8	11	9	10	14
*χ2, p < 0.01; n = 2,107*							

**Table S22.** U.S. concern about who will be impacted by global warming by age category; *How concerned are you about the impact of global warming on…. [all people, all children, your children, people in the U.S., you, your health].[not at all concerned = 1, extremely concerned =7]*

	**National average**	18–29	30–44	45–59	60+
***All people***					
*7*	**24**	24	18	29	26
*6*	**16**	16	18	16	15
*5*	**18**	19	21	17	17
*4*	**19**	18	20	17	20
*3*	**8**	8	8	6	8
*2*	**5**	4	6	5	6
*1*	**9**	11	8	10	7
*χ2, p = 0.028; n = 2,136*					
***All children***					
*7*	**30**	30	26	34	32
*6*	**17**	16	22	15	16
*5*	**17**	17	17	18	16
*4*	**16**	17	16	14	15
*3*	**7**	6	7	5	8
*2*	**5**	5	4	4	6
*1*	**8**	9	9	9	7
*χ2, p = 0.072; n = 2,130*					
***Your children***					
*7*	**30**	30	27	33	29
*6*	**15**	17	19	11	15
*5*	**15**	14	16	14	15
*4*	**15**	16	15	15	15
*3*	**7**	7	6	5	8
*2*	**5**	4	3	6	5
*1*	**14**	12	14	16	11
*χ2, p = 0.016; n = 2,111*					
***People in the U.S.***					
*7*	**21**	20	17	24	23
*6*	**16**	16	15	17	16
*5*	**19**	19	22	17	19
*4*	**21**	21	23	22	19
*3*	**8**	7	9	7	10
*2*	**6**	5	5	5	7
*1*	**9**	12	8	9	7
*χ2, p = 0.060; n = 2,142*					
***You***					
*7*	**21**	23	18	25	19
*6*	**14**	13	13	15	14
*5*	**17**	18	19	15	18
*4*	**21**	21	23	21	19
*3*	**9**	9	9	8	10
*2*	**7**	6	7	7	8
*1*	**11**	10	11	11	12
*χ2, p = 0.343; n = 2,119*					
***Your health***					
*7*	**24**	22	20	28	24
*6*	**15**	17	17	14	14
*5*	**17**	19	16	18	15
*4*	**19**	16	20	19	18
*3*	**9**	10	9	6	11
*2*	**6**	4	7	6	7
*1*	**10**	11	9	9	11
*χ2, p = 0.032; n = 2,136*					
***Your lifestyle***					
*7*	**13**	15	10	15	12
*6*	**10**	9	8	10	11
*5*	**17**	17	17	18	15
*4*	**25**	24	27	25	25
*3*	**12**	9	14	11	13
*2*	**10**	11	9	9	11
*1*	**13**	13	14	12	14
*χ2, p = 0.124; n = 2,126*					
***Your future***					
*7*	**21**	24	18	24	20
*6*	**15**	16	16	16	14
*5*	**19**	20	21	17	16
*4*	**19**	18	18	20	19
*3*	**10**	8	11	8	11
*2*	**6**	4	6	6	10
*1*	**10**	10	9	10	10
*χ2, p = 0.014; n = 2,133*					

**Table S23.** Canadian perceptions of likelihood of increased specific risks by education level; *(Prompted) I will now read you a list health risks that affect many Canadians today. Would you say that* *climate change* *definitely, likely, likely not or definitely does not increase the risk of: (n =  1,600).* **Percent of definitely responses**

	**National average**	Less than high school	High school graduate	Some college	University degree
Respiratory/breathing problems	**46**	51	47	49	42
Sunburn	**46**	55	49	45	44
Heat stroke	**39**	43	42	40	35
Injuries from storms/extreme weather	**32**	41	30	33	30
Cancer	**32**	38	34	31	32
Infectious diseases	**27**	30	31	27	24
*n=*	*1,600*	*168*	*257*	*582*	*577*

**Table S24.** Canadian perceptions of likelihood of increased specific risks by income level; *(Prompted) I will now read you a list health risks that affect many Canadians today. Would you say that climate change definitely, likely, likely not or definitely does not increase the risk of: (n = 1,600).* **Percent of definitely responses**.

	**National average**	Less than $40,000	$40,000–$74,999	$75,000 to $99,999	$100,000+
Respiratory/breathing problems	**46**	51	46	46	37
Sunburn	**46**	49	47	46	36
Heat stroke	**39**	40	40	39	30
Injuries from storms/extreme weather	**32**	36	33	29	25
Cancer	**32**	35	32	31	26
Infectious diseases	**27**	29	28	29	18
*n=*	*1,600*	*392*	*435*	*201*	*299*
